# Do warning calls boost survival of signal recipients? Evidence from a field experiment in a group-living bird species

**DOI:** 10.1186/1742-9994-10-49

**Published:** 2013-08-13

**Authors:** Michael Griesser

**Affiliations:** 1 Anthropological Institute and Museum, University of Zurich, Zurich, Switzerland

**Keywords:** Alarm calls, Vocal communication, Anti-predator signal, Survival

## Abstract

**Introduction:**

Warning calls are a widespread anti-predator adaptation, which can signal unprofitability to predators or alert other potential targets of the predator. Although it is tacitly assumed that the recipients of warning calls experience a reduction in predation risk, this crucial assumption remains untested. Here I tested this hypothesis with a field experiment in the group-living Siberian jay, *Perisoreus infaustus*. I exposed male or female breeding adults that were foraging together with a non-breeder (related or unrelated) to a model of their main predator (goshawk *Accipiter gentilis*) in autumn. I then recorded the warning call response of breeders as well as the reaction time of non-breeders, and followed the subsequent survival of non-breeders until spring.

**Results:**

In most experiments (73%), non-breeders were warned by the more experienced breeders. Warning calls almost halved the reaction time of non-breeders during the experiment and influenced the survival of call recipients: non-breeders that were warned had a higher subsequent survival (19 out of 23) than non-breeders that were not warned (2 out of 5). However, neither kinship, group size, the age of the non-breeder, or the habitat structure of the territory had an influence on the survival subsequent to the experiments.

**Conclusions:**

Since earlier studies showed that breeders are consistent in their warning call investment across different contexts, breeders that did warn non-breeders in the experiment were likely to have done so in subsequent, natural attacks. Consequently non-breeders living with breeders that called had a better chance of surviving predator attacks. Thus, these results suggest that warning calls have the potential to boost the survival of signal recipients, confirming a pivotal, yet hitherto untested assumption of the effect of warning calls.

## Introduction

Warning calls are a widespread anti-predator behaviour [1]. They may be directed at a predator to signal that it has been detected (so called pursuit-deterrent signals [2]), or at other potential targets of the predator [1,3]. Pursuit-deterrent signals reduce the risk of a target being killed during a predator attack when compared to individuals that do not signal [2-4], although this may depend on predator type [3]. Signals given specifically in the presence of conspecifics are likely to be directed at other potential prey. They can convey information about predator identity [5], response urgency [6] or predator behaviour [7]. Such information is likely to enable signal recipients to make appropriate escape decisions [1], and if warning others is a function of these calls, signal recipients should have an enhanced probability of surviving predator encounters. However, although it is tacitly assumed that the recipients of warning calls experience a reduction in predation risk, no study to date has shown that hearing warning calls enhances the recipients’ survival [1], hampering our understanding of the evolution of these widespread adaptations. Moreover, a survival benefit of call recipients is a key assumption of many theoretical models investigating the evolution of alarm calls [1,8], but this assumption remains untested.

Here, I use data from field experiments in the group-living Siberian jay (*Perisoreus infaustus*) to test this hypothesis. Jays live in cohesive groups on year-round territories in boreal forests [9], and aside from the dominant breeding pair, groups can contain retained offspring (young that had remained on their natal territory beyond independence) and/or unrelated immigrants (mean group size = 3.05, range 1–7) [9,10]. Goshawks (*Accipiter gentilis*) are the main predator of jays responsible for 70% of all kills (see below), while owls or pine marten (*Martes martes*) kill jays at a much lower frequency [11]. In response to predators, jays have evolved numerous warning calls which convey information on the risk posed by a predator and the behaviour of their main predator to other group members [7,12]. In particular breeders give warning calls during predator encounters, which are given in the presence of kin and, to a lesser extent, also immigrants [7,12-14].

I examined whether warning calls boost the subsequent survival of call recipients through a single experimental exposure to an attacking goshawk model. I first tested whether warning calls reduce the reaction time of non-breeders (i.e. the time from when the goshawk model emerged from its cover until a non-breeder took off from the feeder). This assumption is crucial given that this would provide a proximate mechanism linking warning calls to an increased likelihood to survive predator ambush attacks. Then, I explored the role of the following factors which could affect non-breeder survival. Non-breeders that receive warning calls during simulated attacks should be more likely to survive subsequent attacks. Given that the main predator of Siberian jays, goshawks, are ambush hunters which rely on surprise attacks [15], being warned in a critical situation is likely to improve the chances of escape from an approaching goshawk. Earlier experiments in this species showed that breeders are nepotistic in their antipredator behaviours, and give warning calls in particular when together with retained offspring [13]. Thus, kinship could influence the likelihood of being warned and thus improve the survival prospects. Predation risk has been shown to depend on the age of an individual (younger, inexperienced individuals have a higher predation risk than older, more experienced individuals [16]) and group size (individuals in larger groups have a reduced risk of being killed by a predator [1]). Moreover, predation risk at the study site is not uniform across the landscape as territories which are less dense offer less protection from hawks [17]. Thus I also explored the role of non-breeder age, group size and territory openness (proportion of unmanaged patches, see below) in the survival of non-breeders.

## Results

**Figure 1:**
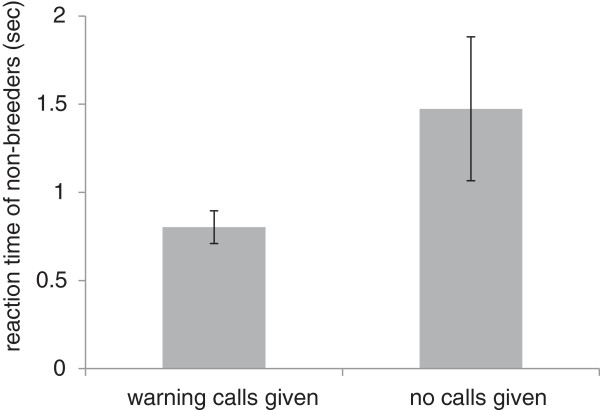
**Effect of warning calls of Siberian jay breeders on the reaction time of non-breeders during simulated goshawk attacks.** Non-breeders which received a warning call (N = 23 experiments) escaped faster to nearby cover than non-breeders which were not warned (N = 8 experiments). Data from two experiments were missing since the camera recording the whole set-up did not work properly.

**Figure 2:**
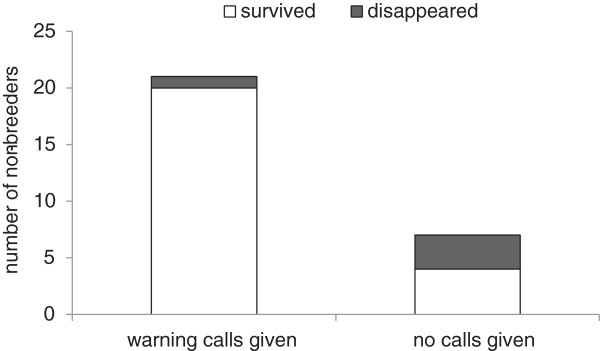
**Influence of warning calls given by Siberian jay breeders during simulated goshawk attacks in autumn on subsequent winter survival of non-breeders (N = 28 individuals).** Non-breeders living with breeders which warn (N = 21 individuals) had a higher over-winter survival than non-breeders which were not warned (N = 7 individuals).

Upon discovering the attacking hawk model, most breeders gave warning calls (24 out of 33 breeders), causing all non-breeders to escape swiftly to nearby cover. Warning calls influenced the reaction time of the non-breeders, which was 46% shorter for non-breeders that were warned compared to non-breeders that were not warned (General Linear Mixed Model, using individual as repeated factor to control for the fact that 5 non-breeders were tested in two different dyads: F = 5.69, p = 0.025; Figure 1). Consequently, non-breeders that had been warned during the attack had a higher probability of surviving the subsequent winter months compared to non-breeders that had not been warned (Figure 2; Logistic Regression: estimate ± SE = 2.17 ± 1.05; Z = 2.06; p = 0.04; Table 1). Model selection procedures showed that the model containing the factor “warning calls” had the highest explanatory degree, and warning calls was the factor with the largest sum of QIC weights (0.83; Table 1). This suggests that warning calls improved the survival of non-breeders.

**Table 1:** Model selection for the factors affecting survival of 28 Siberian jay non-breeders, using logistic regression

**Model**	**QIC**	**ΔQIC**	**Weight**
Warning call	30.73	0	0.24
Warning call + group size	31.72	0.99	0.15
Warning call + habitat	32.92	2.19	0.08
Warning call + habitat + warning call x habitat	33.22	2.49	0.07
Warning call + group size + habitat	33.31	2.58	0.07
Warning call + group size + warning call x group size	33.53	2.8	0.06
Warning call + age	33.65	2.92	0.06
Warning call + group size + age	33.68	2.95	0.05
Warning call + kinship	33.75	3.02	0.05
Group size + age	33.86	3.13	0.05
Age	34.21	3.48	0.04
Kinship	34.44	3.71	0.04
Age + kinship	35.4	4.67	0.02
Group size	35.69	4.96	0.02
**Predictor**	**Sum of weights**		
Warning call	0.83		
Group size	0.40		
Age	0.23		
Habitat	0.22		
Kinship	0.11		

All models with a ΔQIC < 5 are shown. QIC = quasi-likelihood information criterion [34], ΔQIC = difference between focal model and model with lowest QIC value, weight = relative probability of focal model within candidate set. Warning call = warning call heard or not; group size; age: age of non-breeder; habitat = proportion unmanaged forest on territory; kinship: kinship of non-breeder in relation to breeder.

## Discussion

Many prey species are known to give warning calls when encountering predators [1]. A number of field studies have demonstrated that call recipients show appropriate responses to warning calls, such as the silencing calls of white-browed scrubwrens *Sericornis frontalis* parents causing nestlings to remain silent in the presence of a nest predator [18], or nest predator specific warning calls of great tit *Parus major* parents causing nestlings to stay or escape from the nest box depending on the predation threat [19]. When it comes to survival benefits of warning calls however, they only have been assessed for pursuit-deterrent signals directed at predators, which have been shown to reduce the predation risk of callers directly [2,3]. Thus, to my knowledge this is the first study investigating whether warning calls directed at conspecifics can influence their survival, even though it has been implicitly assumed that this is the primary function of warning calls [1,8,20,21]. My results show that Siberian jay non-breeders that are warned during a hawk attack escape faster to nearby cover. Consequently, call recipients have higher survival compared to individuals that are not warned, confirming a pivotal, yet hitherto untested assumption of the effect of warning calls.

Warning calls provide signal recipients in high risk situations (i.e. a goshawk attack) with crucial information, allowing unaware individuals to take appropriate escape measures [5,7]. In Siberian jays and other prey species, attack calls cause signal recipients to escape to nearby cover immediately [1,7,13]. Given that goshawks rely on ambush attacks [15], the early detection of an attacking hawk allows prey to escape successfully [1,22]. Thus, living in a group with a breeder that gives warnings during hawk attacks shortens the reaction time of non-breeders, resulting in an odds ratio of survival that is 47% higher compared to living in a group in which the breeders did not give a warning. While the existence of a survival benefit of receiving warning calls intuitively makes sense, no other study has so far attempted to link warning calls to survival of signal recipients [1]. Further support for a survival benefit of warnings on signal recipients comes from fathead minnows (*Pimephales promelas*), where individuals exposed to alarm pheromones survived longer [23]. In this species, alarm pheromones increase shoaling, which is an efficient antipredator strategy, but their study did not test for a mechanism underlying the improved survival.

Studies investigating warning signals highlighted that calls in several species are directed at kin, implying a kin selected benefit [3,24,25]. However, given that these species live in groups and no dyadic assessment of the effects of alarm calls on survival was made, it remains unclear if warning calls in these systems provide a survival benefit. In the Siberian jay, breeders preferentially warn retained offspring in the presence of predators rather than unrelated immigrants [7,12-14,26], and retained offspring have a higher overwinter survival than immigrants [11]. Moreover, overwinter survival is higher in territories with a high proportion of unmanaged habitat, which provides more protection from visual ambush hunter, such as hawks. However, this study did not find an effect of kinship or habitat structure on survival, which is likely to reflect a small and unbalanced data set with respect to kinship and habitat structure.

A specific assumption of my study is that breeders are consistent in their propensity to warn non-breeders. Preliminary experiments showed that breeders habituated after a single exposure to the attacking hawk model and reduced their willingness to give warning calls, and thus it was not possible to investigate the consistency of individual breeders giving warning calls during hawk attacks. Since the warning calls investment and antipredator behaviours of Siberian jay breeders consistently vary depending on kinship, predator category and risk posed by a predator [12-14,26], it seems reasonable to assume that individual breeders also are consistent in their propensity to warn specific individuals. Alternatively, the correlation between warning call investment of breeders and increased overwinter survival of non-breeders could reflect non-breeder quality, or differences in habitat quality. Winter survival of non-breeders is correlated with the number of fault bars in the feathers, but interestingly, immigrants have fewer fault bars in their feathers than retained offspring [11]. Since female breeders do not warn immigrants [13], it seems unlikely that breeders adjust their investment in warning calls depending on non-breeder quality. Earlier analyses looking into warning call investment did not include habitat quality in the analyses [12-14,26]. However, groups in both low-risk and high-risk territories contained retained offspring and immigrants, and were used for these experiments and this study, reducing the risk of a systematic bias in the data set.

## Conclusions

Studies on warning calls have been important for our understanding of reciprocal altruism, kin selection, and cooperation [8,27,28], as well as the underlying cognitive processes and the evolution of communication, including our own language [8,29]. A key assumption of these studies is that warning calls reduce the predation risk of call recipients, or provide another fitness benefit to the caller [8].While a plethora of studies investigated the proximate factors affecting warning calls [1] and models addressed ultimate factors facilitating their evolution [1,8,21], further studies in other systems are needed to see whether warning calls generally can boost the survival of call recipients.

## Methods

Data for this study were collected in an individually colour-ringed population of Siberian jays that has been studied from 1989 onwards near Arvidsjaur, northern Sweden [9,13]. Here, I use data collected between autumn 1999 and 2000 on 24 territories. A 50 μl blood sample was taken from all individuals for molecular sex determination [30]. Details regarding bird capture, blood sampling and mounting radio transmitters are described in detail in [10] and [13], and all the work was done under the license of the Umeå ethics board (license number A80-99 and A45-04) and the Swedish Bird Ringing Centre (ringing licence number 675).

To investigate whether warning call protection affects survival, dyads of male or female breeders together with either one retained offspring or an immigrant (*n =* 10 experiments each combination, total *n =* 40 experiments) foraging on a feeder were attacked with a life-sized wooden goshawk model. Experiments were only done when other group members were out of sight and thus the decision of the breeder to give a warning call or not should only depend on the presence of the non-breeder on the same feeder. I used two video cameras and direct observations to record the presence of warning calls and the caller’s identity. Each breeder was only exposed once to the model to avoid habituation. Further details of the field methodology are given elsewhere [13].

Survival of non-breeders subsequent to the experiments was assessed using field observation of birds the following March, before the onset of the breeding season. Data from other years showed that all 110 individuals radio-tagged in autumn either stayed on their territory and survived until March (94 individuals), or were found dead on their territory killed by predators (16 individuals killed by goshawks, four by owls, two by pine martens, one by an unidentified aerial predator [11,31], unpublished survival data). Thus, non-breeders which disappeared during winter were almost certainly killed by predators.

I excluded from the analysis four non-breeders that had become breeders before March the following year, given that this meant the group size and social structure of these groups had changed. I also excluded two non-breeders since they were the first to give a warning call and it was therefore impossible to say whether these breeders would have called for the non-breeders. In one group the breeding male was removed as part of another experiment after it had been tested for this study with one retained offspring [31], and therefore this retained offspring was not included in the analyses. Since five non-breeders were exposed to hawks twice in two different dyads (which was also the case for two non-breeders that became breeders, see above), I could link warning call protection to survival in 28 non-breeders (16 retained offspring, 12 immigrants) which lived all winter together with the same breeders as during the experiment.

I assessed the kinship of non-breeders in a group by following reproduction (*n =* 21 individuals) or by observing the behavioural interaction of breeders and non-breeders on feeders (*n =* 7 individuals). In most groups, nests were found by following breeding adults that had been radio-tagged. All nestlings in successful nests were ringed with a metal ring and three colour rings, allowing recognition of individuals as retained offspring if they remained with their parents after independence. In groups where reproduction was not followed, I assessed the aggressive interactions between breeders and unringed non-breeders on feeders following a standard protocol [26]. Breeders are rarely aggressive towards retained offspring, while they frequently displace or chase away unrelated non-breeders from the feeder [9,26]. Assessing relatedness using this behavioural proxy has been shown to be reliable when compared with individuals of known relatedness [26].

An earlier study showed that habitat structure influences survival of Siberian jays [11], and thus I included the proportion of unmanaged patches within each territory into the analyses. Forests at the study site cover a gradient from intensely managed patches to pristine forests [32]. In managed patches, the entire understory (small spruces, deciduous trees) is removed every 20–40 years to enhance timber production [32]. These patches are therefore more open and provide less visual cover than unmanaged patches, facilitating prey detection for predators [17]. I measured the proportion of unmanaged patches within each territory in the field with a GPS receiver or from aerial images (see [17] for a detailed description of the field methodology).

I analysed the data using SAS 9.3 (SAS institute, Cary, North Carolina). To analyse the effect of independent factors that influenced non-breeder survival, I used logistic regressions with a binomial error distribution using the GENMOD procedure, using an information criterion approach to find the models with the highest explanatory degree [33]. Given that five non-breeders were both exposed with the male and female breeder of their group, I included non-breeder identity in the repeated statement. Since these models are quasi-likelihood based, it is not possible to calculate the Akaike Information Criterion (AIC), but instead the quasi-likelihood information criterion (QIC) is used [34]. The best model was defined as the one with the lowest QIC value, while models with a ΔQIC larger than 5 were considered as unlikely and therefore excluded from the final model set [33]. I used all possible combinations of the following variables in the analyses: if a warning call was given or not, kinship, age of the non-breeder, group size, and habitat structure of the territory, as well as possible interactions of the independent factors.

## Competing interests

The author declares that he has no competing interests.
